# Goodwyn’s Compound Syrup of the Hypophosphites

**Published:** 1884-12-20

**Authors:** 


### GOODWYN'S COMPOUND SYRUP OF THE HYPOPHOSPHITES.

The formula of the above preparation, as it appears in an advertisement in this journal, must strike every intelligent physician with favor. This addition of a minute quantity of arsenic and strychnia, with pepsin, to the other standard ingredients of the hypophosphite combinations, is certainly a happy conception, and gives to the preparation a wide therapeutical application. We commend the article to our readers as one well conceived, neatly put up, and by a pharmaceutist worthy of the confidence of the profession.

